#  Effects of Hyul-Bu-Chuke-Tang on Erythrocyte Deformability and Cerebrovascular CO_**2**_ Reactivity in Normal Subjects

**DOI:** 10.1155/2012/725241

**Published:** 2012-05-28

**Authors:** Woo-Sang Jung, Joo-Young Park, Hyung-Sik Byeon, Young-Jee Kim, Jung-Mi Park, Seong-Uk Park, Seung-Yeon Cho, Sang-Kwan Moon

**Affiliations:** Department of Cardiovascular and Neurologic Diseases, College of Korean Medicine, Kyung Hee University, Seoul 130-702, Republic of Korea

## Abstract

*Aim*. Hyul-bu-chuke-tang (HCEt) is a well-known traditional herbal medicine that is used for the treatment of ischemic cerebrovascular disorders. We investigated the acute effects of HCEt on erythrocyte deformability and cerebrovascular CO_2_ reactivity (CVR) in healthy male subjects. *Materials and Methods*. We examined erythrocyte deformability in an HCEt group (*n* = 14) and a control group (*n* = 10). CVR was measured using hyperventilation-induced CO_2_ reactivity of the middle cerebral artery and transcranial Doppler (TCD) in the HCEt group (*n* = 11). A historical control group (*n* = 10) of CVR measurements was also created from our previous study. All measurements were performed prior to and 1, 2, and 3 hours after HCEt administration. *Results*. HCEt significantly improved erythrocyte deformability 1 hour after administration compared to the control group (2.9 ± 1.1% versus −0.6 ± 1.0%, *P* = 0.034). HCEt significantly improved the CVR 2 hours after administration compared to the historical control group (9.1 ± 4.0% versus −8.1 ± 4.1%, *P* = 0.007). The mean blood pressure and pulse rate did not vary from baseline values in either group. *Conclusions*. We demonstrated that HCEt improved erythrocyte deformability and CVR. Our findings suggest that an improvement in erythrocyte deformability contributes to HCEt's effect on cerebral microcirculation.

## 1. Introduction

Traditional herbal medicine is widely used in Asia to optimize the treatment of cerebrovascular disease with conventional therapy [1]. Hyul-bu-chuke-tang (HCEt, known as Xue-fu-zhu-yu-tang in Chinese) is one of the best-known traditional herbal medicines for the treatment of cerebral infarction in Korea. The therapeutic effect of HCEt on ischemic vascular diseases has been verified recently [2, 3]. However, HCEt is a complex of 11 medical plants, and its therapeutic mechanism is likely complicated.

Traditional Chinese Medicine suggests that HCEt impacts blood stasis syndrome, which is a pathological state of blood stagnancy in a certain area of the body [4]. Impairments in hemorheology and microcirculation play important roles in the pathophysiology of blood stasis syndrome, which is consistent with TCM theory [5, 6]. HCEt reduces platelet aggregation and enhances erythrocyte deformability and blood filtration rates *in vivo* [7]. Erythrocyte deformability is an important factor for microvasculature perfusion [8]; diminished erythrocyte deformability increases microcirculatory resistance [9]. Hemorheological factors modify blood fluidity and blood flow behavior [5]. However, the effects of HCEt on erythrocyte deformability and cerebral microcirculatory blood flow in humans have not been researched extensively.

The present study investigated the acute effects of HCEt on erythrocyte deformability in normal subjects. We also measured cerebrovascular CO_2_ reactivity using transcranial Doppler ultrasonography (TCD) to preliminarily investigate the effect of HCEt on cerebral microcirculation.

## 2. Materials and Methods

Two consecutive investigations were included in our study. The first series measured erythrocyte deformability following HCEt administration using a microfluidic ektacytometer in 24 healthy subjects. The second series evaluated the CVR following HCEt administration using TCD in 11 healthy subjects.

### 2.1. Subjects

Thirty-five healthy male volunteers (mean age: 27.1 ± 0.5 (S.D) years)) participated in the study. The Institutional Review Board at the Kyung Hee Medical Centre approved this study, and all participants signed written consent forms. None of subjects had a history of neurological disorders, such as stroke, head injury, psychiatric disorders (e.g., mental retardation, schizophrenia, and depression), hypertension, diabetes mellitus, drug abuse, alcohol dependence/abuse, or a disease or previous surgery that could influence drug absorption. The subjects abstained from smoking and drinking alcohol, coffee, or tea for 12 hours prior to examination. 

### 2.2. Preparation of Hyul-Bu-Chuke-Tang (HCEt)

The Department of Preliminary Pharmaceutical Preparation of the Kyung Hee University Korean Medical Centre (KHUKMC) synthesized the HCEt. HCEt was prepared as dry extract granules, 6 g per pouch, which contained the 11 species of medicinal herbs in Table 1. Kim in the Department of Pharmaceutics at KHUKMC identified the plant materials, and voucher specimens (number 10-10-03) were deposited in the Herbarium of the Department of Pharmaceutics at KHUKMC. Crude herbs (*Persicae Semen *424 g, *Angelical Gigantis Radix *320 g, *Gehmannial Rhizoma *320 g, *Carthami Flos *320 g, *Achyranthis Bidentatae Radix *320 g, *Aurantii Fructus *216 g, *Paeoniae Radix Rubra *216 g, *Platycodi Radix *216 g, *Cnidii Rhizoma *160 g, *Bupleuri Radix *160 g, and * Glycyrrhizae Radix *160 g; *total herbs *2832 g) were cut into small pieces, and the herb mixture was extracted in a reflux condenser for 3 h with 20.000 mL of hot water. The solution was filtered through filter paper (Whatman no. 1) and concentrated using a spray drying process (drug-extract ratio: 3.75 : 1). The dry extracts were granulated using 3 different binders, lactose (200 g), polyvinylpyrrolidone (PVP, 160 g), dextrin (300 g) and ethanol (1400 mL). The ethanol was evaporated after the binding procedure.

### 2.3. Measurement of Erythrocyte Deformability

We examined erythrocyte deformability in the HCEt group (14 healthy males, mean age: 28.9 ± 3.4 (S.D) years) and the control group (10 healthy males, mean age: 27.3 ± 1.3 years). The HCEt extract was administered orally at 8 am. The measurements were performed prior to and 1, 2, and 3 hours after HCEt administration. Four blood samples were obtained from each subject. The control group did not receive treatment.

A Rheoscan-D microfluidic ektacytometer (Rheo Meditech, Seoul, Korea) measured erythrocyte deformability. One drop of blood was obtained from each volunteer's fingertip using a finger prick (Seahan Medical, Seoul, Korea). The erythrocyte suspension was prepared by mixing 6.0 *μ*L whole blood and 0.5 mL of a highly viscous PVP solution (31 mPa) in phosphate-buffered saline (0.14 mM). A 0.5-mL aliquot of the erythrocyte suspension was placed in the test chamber of a disposable kit, which included a microchannel (Rheo Meditech, Seoul, Korea). Differential pressure drove the erythrocyte suspension through the microchannel (0.2 × 4 × 40 mm) of the disposable kit, and the waste was collected in a waste chamber. A laser beam (635 nm wavelength) from a 1.5-mW laser diode passed through the diluted erythrocyte suspension during the flow. The diffraction pattern of the moving erythrocytes at plural shear stresses was projected onto a screen, and the images were captured by a CCD-video camera every 0.5 sec. The images were analyzed using an ellipse-fitting computer program. The average shear stress ranged from 0 to 30 Pa. The elongation index (EI) of erythrocyte deformability was defined as follows [10]: EI = (*L* − *W*)/(*L* + *W*), where *L* and *W* are the major and minor axes of the erythrocyte ellipse, respectively. The microchannel was discarded after each measurement.

### 2.4. Measurement of Cerebrovascular CO_2_ Reactivity (CVR) Using Transcranial Doppler Ultrasonography

We investigated cerebrovascular reactivity using TCD. CVR was measured using hyperventilation-induced carbon dioxide reactivity of the middle cerebral artery in 11 healthy young male volunteers (mean age: 24.8 ± 1.1 years) in the HCEt group. All participants received 1 pouch of HCEt extract at 8 a.m. TCD was performed prior to HCEt administration and 1, 2, and 3 h after HCEt administration.

CVR was assessed during 1-min hyperventilation-induced hypocapnia, which is similar to a previous TCD study that used a Multi-Dop X4 system (Compumedics DWL, Singen, Germany) [11, 12]. Each subject was examined in the supine position. The 2-MHz-pulsed Doppler probe was placed on the temporal region (ultrasonic window). A removable bilateral probe holder (LAM-Rack; Compumedics DWL) was used to avoid probe shifting and permit continuous measurement. All measurements commenced after the subjects had stabilized (approximately 5 min). The mean blood flow velocity of the middle cerebral artery was calculated continuously as the time-averaged maximum velocity over the cardiac cycle, which was computed from the envelope of the maximum frequencies. CVR was determined as the percent change in mean blood velocity per change of *P*_ETCO_2__ as calculated by the following formula [13]:

(1) $${\text{CO}}_{2}\;\text{reactivity}=100\times \frac{\left[V_{\text{rest}}-V_{\text{hypocapnia}}\right]/V_{\text{rest}}}{{\Delta P}_{{\text{ ETCO}}_{2}}},$$

where *V*_rest_ is the mean blood velocity in the normocapnic condition for 5 min prior to the initiation of hyperventilation. *V*_hypocapnia_ is the mean blood velocity of the latter half of the 1 min-hyperventilation period, and Δ*P*_ETCO_2__ (partial pressure of end-tidal carbon dioxide; a measure of the amount of carbon dioxide in the exhaled air) is the change in  *P*_ETCO_2__ from baseline to maximal hyperventilation. CO_2_ reactivity was expressed as %/min⁡. 

We recorded blood pressure, heart rate, and *P*_ETCO_2__ simultaneously using a Cardiocap S/5 capnometer (Datex-Ohmeda, Helsinki, Finland) to monitor the covariates that may regulate cerebral artery blood flow. Blood pressure and pulse rate were measured under stable normocapnic conditions prior to hyperventilation. These measurements were performed 4 times at 2-min intervals to determine the mean blood pressure. An oxymetry apparatus on the subject's finger simultaneously monitored the pulse rate. A Cardiocap S/5 collector-connected nasal prong monitored *P*_ETCO_2__, and each subject only breathed through the nose during the study. The Cardiocap S/5 collector software program calculated the mean pulse rate and *P*_ETCO_2__ at certain timepoints during the assessment.

A historical control group that included placebo control data on TCD-measured CVR was also created from our previous study [11]. We previously investigated CVR in 2006 in 10 healthy young male volunteers (age: 26.1 ± 1.8 years) who had received a placebo control drug using an identical method and device as the current study. 

### 2.5. Statistical Analysis

We utilized the Statistical Package for Social Science version 12.0 for Windows (SPSS, Chicago, IL). Data are summarized as the means ± standard deviation or means ± standard mean error. *Paired t-*test-compared variables prior to and after administration in each group. *Independent t-*test-compared variables in the HCEt and control groups. A *P* < 0.05 was considered significant.

## 3. Results

**Figure 1:**
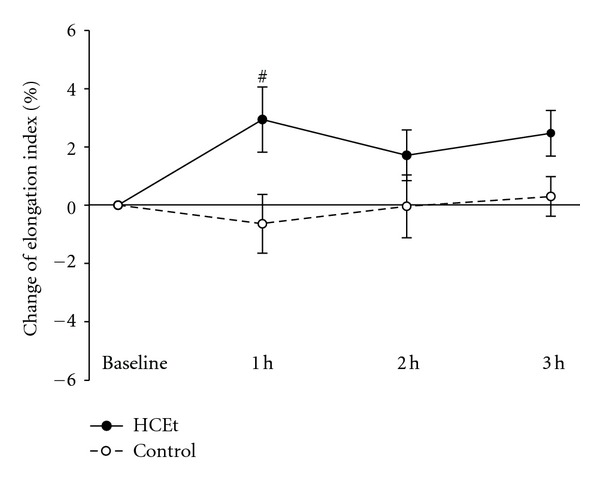
Change of erythrocyte deformability in the HCEt group (*n* = 14) and the control group (*n* = 10) at each time point. All values are the percent change compared to baseline. The vertical bars represent the means ± S.E.M. The *P* values were obtained from *independent t-test*. HCEt: hyul-bu-chuke-tang; h: hour. ^#^ *P* < 0.05 compared to the control group.

The index of erythrocyte deformability (EI) at 1, 2, and 3 h was significantly greater than baseline in the HCEt group (Figure 1). The EI in the control group was not altered. HCEt administration significantly improved erythrocyte deformability after 1 hour compared to the control group (2.9 ± 1.1% (S.E.M) in 14 subjects versus −0.6 ± 1.0% in 10 subjects: 95% confidence interval for difference = 0.3–6.8%, *P* = 0.034). No difference in age between the two groups was observed.

HCEt increased CVR above baseline values at 1 and 2 h after administration. A comparison of the CVR data in the HCEt group to the historical control group using *Student's t-test* revealed significant improvement in the HCEt group (9.1 ± 4.0% (S.E.M) in 11 subjects versus −8.1 ± 4.1% in 10 subjects: 95% confidence interval for difference  = 5.2–29.4%, *P* = 0.007). No difference in age between the two groups was observed.

The erythrocyte deformability (*n* = 14) and CVR (*n* = 11) data of individuals in the HCEt group are shown in Figures 3(a) and 3(b).

 The mean blood pressures and pulse rates did not vary significantly from baseline values during the 3-hour TCD procedure in either group (Table 2). 

## 4. Discussion

This study demonstrated that HCEt improved erythrocyte deformability and increased CVR in young healthy subjects. Our results suggest that HCEt exhibits acute effects on cerebral microcirculation and that HCEt diminished blood flow resistance in distal vessels by improving erythrocyte deformability, which contributed to the increase in CVR.

We demonstrated that HCEt reduced microcirculatory resistance by improving erythrocyte deformability. Erythrocytes must deform to enter microvessels, such as capillaries, which maintain a smaller diameter than erythrocytes [8]. An increase in erythrocytes deformability eases the passage through a capillary, which increases the number of perfused capillaries in the vascular bed, (i.e., capillary recruitment) [14]. The improvement in blood flow was due to an increase in capillary recruitment [15]. Alterations in erythrocyte deformability primarily influence microcirculation resistance in vessels with dimensions that are similar to erythrocyte size (approximately 7-8 *μ*m) [16].

**Figure 2:**
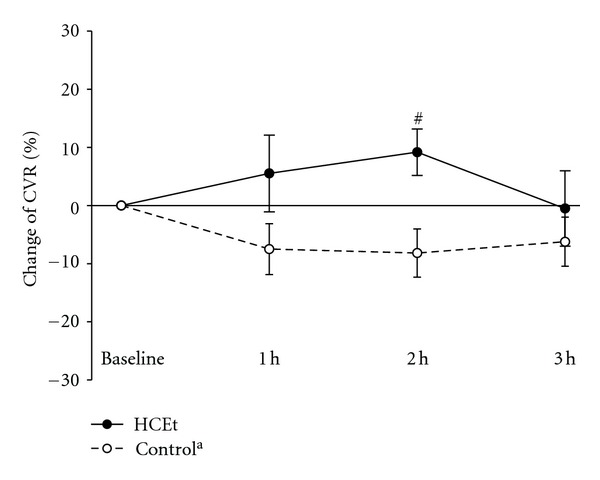
Change of cerebrovascular CO_2_ reactivity (CVR) in the HCEt group (*n* = 11) at each time point. ^a^For comparison, CVR data are also shown for historical control group of 10 healthy young male subjects matched with for age and who received placebo. All values are the percent change compared to baseline. The vertical bars represent the means ± S.E.M. The *P* values were obtained from *independent t-test*. HCEt: hyul-bu-chuke-tang; h: hour. ^#^ *P* < 0.05 compared to the historical control group.

We also confirmed a 9.1% increase in CVR for 2 hours after HCEt administration, which was significant compared to baseline (Figure 2). This result suggests that HCEt increased regional resting cerebral blood flow. CVR is the change in cerebral blood flow velocity in response to changes in *P*_CO_2__, and it is a reliable index of relative changes in cerebral blood flow [17–20]. Cerebrovascular CO_2_ reactivity (CVR) reflects the consequent response of arterioles in the cerebral vascular bed to the dilatory CO_2_ stimulus [13]. Ackerman demonstrated that CVR is proportional to the regional resting blood flow/blood pressure ratio and determined this ratio as conductance (i.e., the reciprocal of cerebrovascular resistance) [17, 20]. We continuously monitored blood pressure and heart rate during CVR examinations and confirmed that these values were constant (Table 2). Therefore, increases in CVR after HCEt administration were proportional to the increase in regional resting blood flow [20, 21]. We postulated that HCEt increased regional resting blood flow as a result of reduced cerebral microcirculatory resistance. 

**Figure 3:**
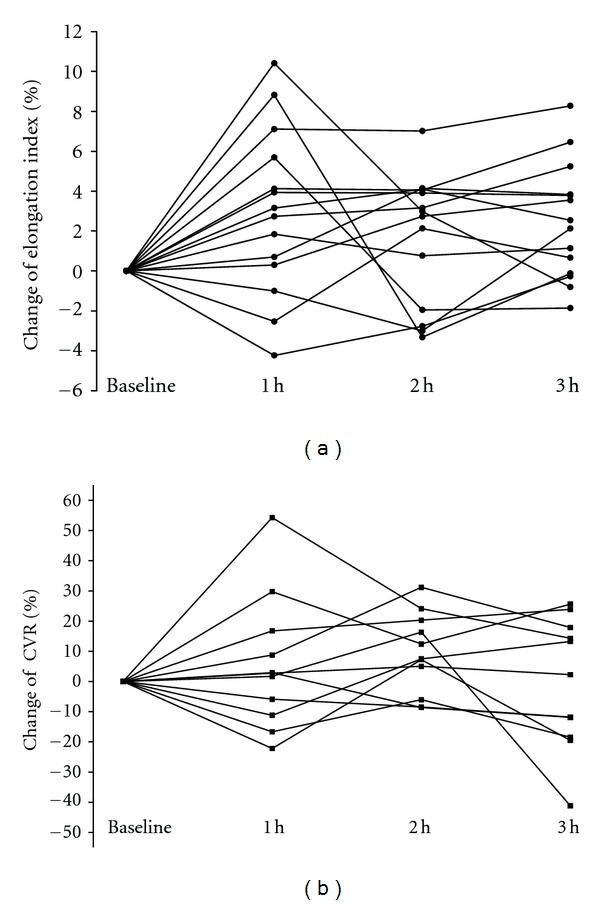
The erythrocyte deformability (a) and cerebrovascular CO_2_ reactivity (b) over time in each individual who received HCEt. All values are the percent change compared to baseline. h: hour; CVR: cerebrovascular CO_2_ reactivity.

This study established the efficacy of HCEt on erythrocyte deformability in normal human subjects for the first time. We also demonstrated that improved erythrocyte deformability contributed to the effect of HCEt on cerebral microcirculation. However, the relationship between the HCEt-induced hemorheological effect and microcirculatory resistance reduction should be interpreted with caution. Individual data changes were dynamic for 3 hours. The time to maximum HCEt effect was not consistent in either parameter in each subject. We must consider that a vasodilatory effect of HCEt directly contributed to the reduction in blood flow resistance. The vasodilatory effect of HCEt has been the focus of previous research. HCEt increases NO production in TNF-r-treated vascular smooth muscle cells from rat aorta, elevates serum NO levels and the NO synthase system in swine after acute myocardial infarction, and decreases serum levels of asymmetric dimethylarginine (ADMA), a nitric oxide inhibitor, in atherosclerotic rabbits [22–24]. However, we suggest that the vasodilatory effect and rheological behavior acted simultaneously due to NO regulation by HCEt. Figures 3(a) and 3(b) illustrate that the distribution patterns of variables between erythrocyte deformability and CVR were remarkably similar among individuals for 2 hours. Externally generated NO also increases erythrocyte deformability in healthy male volunteer blood samples [25]. The antioxidant defense mechanism determines the grade of RBC structural rigidity [26] because the RBC membrane is rich in polyunsaturated fatty acids, which creates susceptibility to oxidative damage [27]. The petals of *Carthamus tinctorius *L. (Safflower) of HCEt exhibits a protective effect on oxygen-free radical-induced oxidative damage to erythrocyte membranes, inhibits platelet and erythrocyte aggregation, and famously promotes blood circulation [28, 29]. *Achyranthes japonica Radix* impacts antioxidant and fibrinolytic effects, and it is used for blood stasis in the peripheral circulatory system [30, 31]. The dried rhizomes of* Cnidium officinale* are widely used for blood circulation due to its free radical-scavenging activities [32, 33]. More studies on each herbal element of HCEt have been performed in the search for improved blood circulation. *Persicae semen* exhibits anticoagulation and thrombotic effects [34, 35]. *Paeonia lactiflora* inhibits thrombosis and platelet aggregation, increases fibrinolytic activity, and exhibits an antioxidant effect [36, 37].

Our results are consistent with previous HCEt studies in the cerebrovascular system. One decoction of Xue-fu-xhu-yu (HCEt) increases vertebral basilar artery blood flow velocity and decreases pulsality index in patients with sudden deafness [38]. Lee et al. reported that this decoction markedly potentiated the recombinant tissue plasminogen activator rt-PA-mediated reduction in infarct volume in a cerebral ischemic region [3]. Our results regarding the effect of HCEt on CVR supports the result of Lee et al. because the increase in CVR represents an improvement in cerebrovascular reserve capacity. Therefore, a decrease in cerebral blood perfusion can be counterbalanced by a reduction in cortical vessel resistance to maintain a sufficient blood supply in the brain [19, 39].

One limitation of the present research is the comparison of current TCD data to historical control data. The potential bias and compounding factors were minimized as much as possible despite the limitations of the designed protocols. The inclusion criteria for subjects and the methods and devices of TCD examination were identical in the previous and current studies. The effects of HCEt on both parameters may appear subject-specific (Figure 3) because TCM exhibits differences in individual drug responses. Basically, HCEt was created for patient with blood stasis syndrome based on the TCM pattern identification theory. Further research of HCEt should be performed in categorized patients using a blood stasis syndrome score.

## 5. Conclusions

We demonstrated that HCEt improved CVR and erythrocyte deformability. Our results suggest that HCEt increases blood flow in the cerebral microcirculation by enhancing erythrocyte deformability. This study provides a basis for further studies on the effect of HCEt in patients with cerebral infarction, especially patients with reduced erythrocyte deformability and impaired cerebrovascular reactivity.

## Figures and Tables

**Table 1:** Constituents of hyul-bu-chuke-tang.

Components	Part used	Percentage
*Angelica acutiloba* Kitagawa (Umbelliferae)	Root	13.3
*Prunus persica* (L.) Batsch (Rosaceae)	Seed	17.7
*Rehmannia glutinosa* Liboschitz (Scrophulariaceae)	Root	13.3
*Carthamus tinctorius* L. (Compositae)	Flower	13.3
*Achyranthes japonica* (Miq.) Nakai (Amaranthaceae)	Root	4.4
*Citrus aurantium* L. (Rutaceae)	Fruit	8.8
Paeonia lactiflora Pall. (Ranunculaceae)	Root	8.8
*Platycodon grandiflorum* (Jacq.) A. DC. (Campanulaceae)	Root	8.8
*Cnidium officinale* Makino (Umbelliferae)	Root	6.6
*Bupleurum falcatum* Linne (Umbelliferae)	Root	2.2
*Glycyrrhiza glabra* L. (Leguminosae)	Root	2.2

**Table 2:** Mean blood pressures and pulse rates during the TCD examination.

		Baseline	After administration		
			1 h	2 h	3 h
Mean BP (mmHg)	HCEt (*n* = 11)	84.7 ± 5.8	84.9 ± 4.1	84.6 ± 5.8	83.6 ± 4.4
	Control (*n* = 10)	85.8 ± 6.5	89.5 ± 6.0	85.7 ± 7.5	86.9 ± 7.7

Pulse Rate (bpm)	HCEt (*n* = 11)	66.4 ± 9.2	65.2 ± 7.2	65.2 ± 7.2	65.5 ± 7.7
	Control (*n* = 10)	71.0 ± 8.7	66.7 ± 9.6	64.1 ± 9.4	64.1 ± 9.7

The data are presented as the means ± standard deviation; no significant difference between baseline and 1, 2, and 3 h values was detected by *paired t-test*. HCEt: hyul-bu-chuke-tang; BP: blood pressure; bpm: beats per minute; h: hour.
